# Advances in Engineered Liver Models for Investigating Drug-Induced Liver Injury

**DOI:** 10.1155/2016/1829148

**Published:** 2016-09-20

**Authors:** Christine Lin, Salman R. Khetani

**Affiliations:** ^1^School of Biomedical Engineering, Colorado State University, Fort Collins, CO 80523, USA; ^2^Department of Bioengineering, University of Illinois at Chicago, Chicago, IL 60607, USA

## Abstract

Drug-induced liver injury (DILI) is a major cause of drug attrition. Testing drugs on human liver models is essential to mitigate the risk of clinical DILI since animal studies do not always suffice due to species-specific differences in liver pathways. While primary human hepatocytes (PHHs) can be cultured on extracellular matrix proteins, a rapid decline in functions leads to low sensitivity (<50%) in DILI prediction. Semiconductor-driven engineering tools now allow precise control over the hepatocyte microenvironment to enhance and stabilize phenotypic functions. The latest platforms coculture PHHs with stromal cells to achieve hepatic stability and enable crosstalk between the various liver cell types towards capturing complex cellular mechanisms in DILI. The recent introduction of induced pluripotent stem cell-derived human hepatocyte-like cells can potentially allow a better understanding of interindividual differences in idiosyncratic DILI. Liver models are also being coupled to other tissue models via microfluidic perfusion to study the intertissue crosstalk upon drug exposure as in a live organism. Here, we review the major advances being made in the engineering of liver models and readouts as they pertain to DILI investigations. We anticipate that engineered human liver models will reduce drug attrition, animal usage, and cases of DILI in humans.

## 1. Introduction

Drug-induced liver injury (DILI) is a leading cause of pharmaceutical attrition and acute liver failures in the US [1]. In particular, DILI has been linked to almost 1000 marketed drugs [2]. DILI can mimic many forms of acute or chronic liver diseases, such as necrosis (cellular death), hepatitis (inflammation), cholestasis (reduction or stoppage of bile flow), fibrosis (scarring), or a mixture of different injury types. While some drugs cause DILI that is predictable and dependent on the dose of the drug (i.e., acetaminophen), many cases of DILI are termed “idiosyncratic” since overt liver injury occurs unpredictably in a small number of patients potentially due to other factors such as environmental stimuli, coadministered drugs, and host risk factors (i.e., age, sex, preexisting disease, and genetics). Idiosyncratic DILI can be mediated by the innate and adaptive immune systems that are triggered by injury to hepatocytes or other cell types of the liver. Ultimately, the interplay between hazardous and adaptive cellular responses can determine whether the liver of a particular patient adapts following a mild injury or proceeds to severe injury due to a drug. In order to mitigate the risk of DILI (and toxicities to other types of tissues), regulatory agencies require testing on live animals before a drug candidate can proceed to human clinical trials. However, animal testing is only 50% predictive of human DILI, likely due to the significant differences in drug metabolism pathways between the livers of animals and humans [3]. In addition, use of young animals with limited genetic diversity under well-defined nutritional conditions for drug safety assessment does not capture the aforementioned host risk factors present in humans. Therefore, given the challenges with screening drugs in animals, regulatory agencies and the pharmaceutical industry are under increased pressures to develop and adopt human-relevant methods to evaluate drug safety prior to exposing live patients to drugs.

In the case of the liver, several different model systems have been developed to provide human-specific data on drug behavior [4]. These include microsomes, cancerous/immortalized cell lines, isolated primary human liver cells, liver slices, and humanized rodents. While these models have already been used in some instances to mitigate the risk of DILI during drug development, there remains a need for model systems that are better predictive of clinical outcomes, with respect to the type and severity of DILI, and can be used to elucidate interindividual variability in drug outcomes. Furthermore, how metabolism in the liver affects toxicity in other tissue types needs to be investigated further using newer culture platforms that link tissue types together through the exchange of culture medium [5].

A spectrum of human liver model systems is being developed to address the above mentioned challenges using engineering tools (i.e., micropatterning, microfluidics, and biomaterials) that enable greater control over the cellular microenvironment to influence cell functions* in vitro*. Here, we will describe the most recent advances in engineered human liver models that have utility for early DILI prediction and to obtain a better understanding of the diverse mechanisms underlying different forms of DILI. We begin with a brief description of conventional/traditional culture models and then discuss engineered liver models starting with static micropatterned cocultures (2D), followed by static spheroids and bioprinted liver models (3D). We then discuss perfused culture platforms that are more technologically complex than static plate-based systems but can be adapted to both 2D and 3D cultures. Precision cut liver slices and humanized liver rodent models are discussed as the most complex and* in vivo*-like liver models currently available. High content readouts and* in silico* predictions are briefly discussed as they pertain to DILI detection. Finally, Section 10 summarizes the key trends as well as the pending issues/questions in the field. We highlight key published studies that demonstrate the different types of model systems and data sets generated for detection of DILI, while referring the reader to other review articles that provide more comprehensive information on specific technologies and/or methodologies.

## 2. Conventional Cultures

Culture of hepatic cell lines and primary hepatocytes on adsorbed or gelled extracellular matrix (ECM) has been carried out for several decades and other reviews cover the genesis and development of this field [6]. Here, we briefly summarize the key conventional/traditional model systems that are useful for assessing the liver toxicity potential of pharmaceuticals. For instance, cancerous hepatic cell lines (i.e., HepG2, HepaRG) in 2D monolayers are widely used for evaluating the toxicities of candidate compounds, especially in early stages of drug development [7]. In some cases, such cell line cultures also enable the study of drug-induced lipid accumulation (i.e., steatosis) [8] and alterations in bile canaliculi dynamics [9], which constitute alterations in hepatic functions that can ultimately lead to liver injury. While cancerous/immortalized hepatic cell lines provide for nearly inexhaustible sources of liver cells for early drug screening, they have some limitations for accurately modeling complex physiological outcomes. In particular, cell lines are ultimately limited to single donors (whereas DILI can vary across multiple individuals), known to display abnormal morphology and levels of liver functions [10], and not always highly sensitive for detection of DILI [11], at least in 2D monolayers.

In contrast to hepatic cell lines, primary hepatocytes, especially from humans, can vary in phenotype across donors and are not always readily available due to sourcing limitations; however, they are the closest representation of human liver physiology if cultured appropriately* in vitro* [6]. Primary hepatocytes in suspension can only be incubated with drugs for 4–6 hours, thereby requiring very high doses of drug to cause any cellular toxicity. Confluent monolayers of hepatocytes adhered to adsorbed collagen can be incubated with drugs for 4–72 hours, but drug metabolism capacities in such cultures are known to show severe downregulation, which negatively impacts correlation with clinical DILI outcomes [12]. Over the years, investigators have devised non-engineering-based techniques to slow down such downregulation. For instance, overlaying confluent hepatocyte monolayers with an ECM gel, such as Matrigel or collagen, can slow down the functional decline of hepatocytes [13]; however, within the first few days, hepatocyte functions in such ECM-overlay culture models still decline to levels that are <10% of those measured in freshly isolated hepatocytes [14]. Culturing hepatocytes in spheroids with tight cell-cell interactions is another technique to mitigate the rapid loss of hepatic functions; however, creating spheroids in a random configuration (such as via nonadhesive plates) leads to nonhomogenous diameters and necrosis in the interior of large spheroids (>200–300 *μ*m). Coculturing with both liver- and non-liver-derived stromal cells has been long known to keep hepatocytes functional for prolonged times as compared to pure monolayers on adsorbed collagen [15]. However, randomly distributed cocultures of hepatocytes and stromal cells display inherent instability in functions over time due to areas of the monolayer that contain suboptimal homotypic and heterotypic cell-cell interactions [14]. Thus, while the aforementioned techniques constitute advances in hepatocyte culture, they are not sufficient to fully stabilize the hepatic phenotype to allow for significant improvements in the prediction of clinical DILI. As we show in subsequent sections, engineering-based tools can augment the techniques discussed above such that the microenvironment around cells is controlled to an extent where functions are better stabilized in a reproducible manner across many experiments and donors so as to be useful for screening drugs with improved prediction of clinical DILI.

## 3. Micropatterned Cocultures

**Figure 1:**
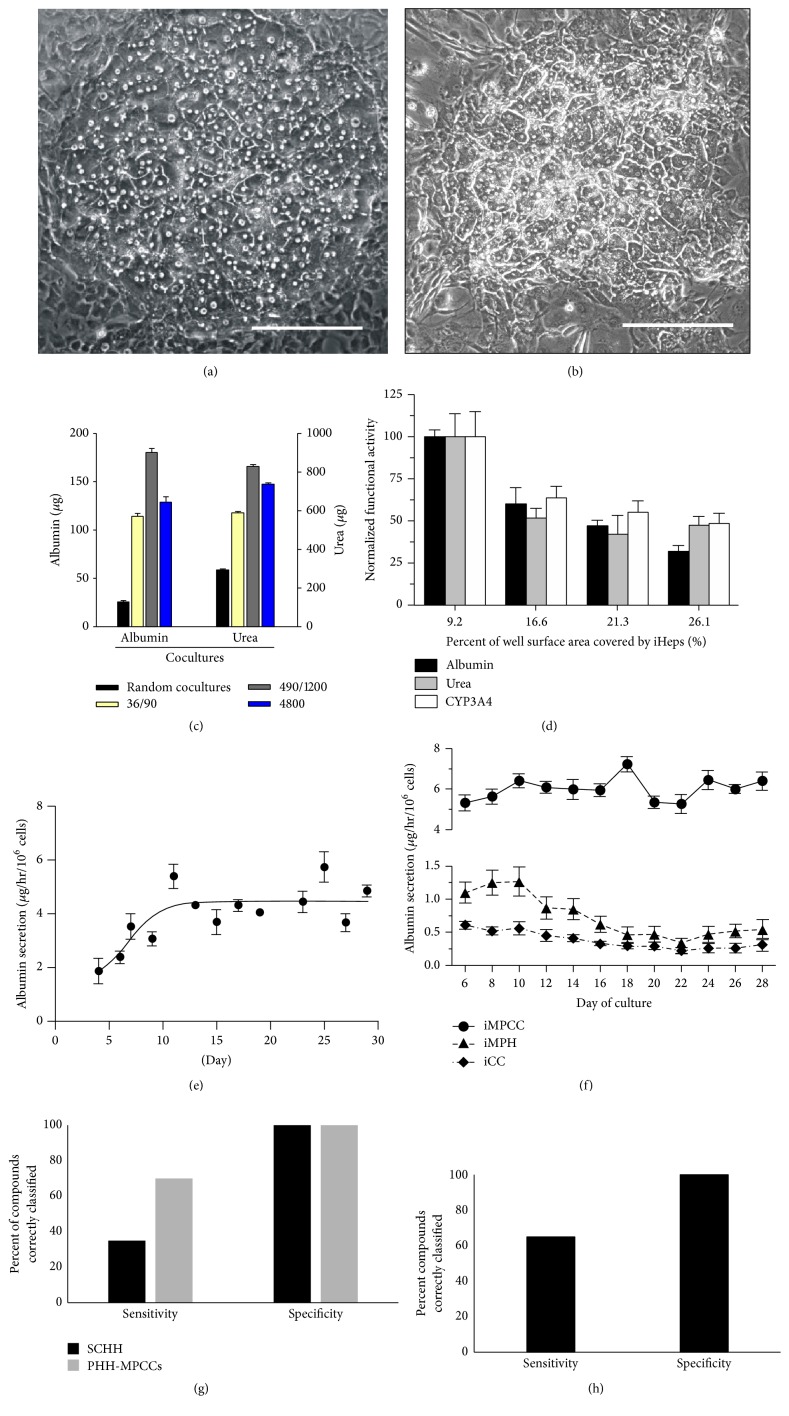
Micropatterned cocultures (MPCCs) containing primary human hepatocytes (PHH) or induced pluripotent stem cell-derived hepatocyte-like cells (iPSC-HH) with supporting 3T3-J2 fibroblasts. Phase contrast images of PHH-MPCCs (panel (a)) and iPSC-HH-MPCCs (panel (b)) display similar hepatic morphology with polygonal shape, formation of bile canaliculi, and distinct nuclei/nucleoli. Scale bars on images represent ~250 *µ*m. The architecture (island diameter, center-to-center spacing, percent of a well's surface area covered by hepatocytes) affects functions in both PHH-MPCCs (panel (c)) and iPSC-HH-MPCCs (panel (d)). In panel (c), cell numbers and ratios were kept constant while changing the diameter (first number) and center-to-center (second number) spacing of the PHH colonies [14]. In panel (d), total well surface area covered by the iPSC-HHs (also called iHeps) was modulated by changing the island diameter and spacing [116]. Albumin secretion levels can be maintained for at least ~1 month in PHH-MPCCs (panel (e)) [117] and iPSC-HH-MPCCs (circles in panel (f), triangles: micropatterned iPSC-HHs without 3T3-J2 fibroblasts, diamonds: iPSC-HH conventional confluent cultures) [116]. Compared to ECM sandwich-cultured primary human hepatocytes (SCHH), both PHH-MPCCs (panel (g)) and iPSC-HH-MPCCs (panel (h)) display higher sensitivity and similar specificity for drug toxicity screening when cultures were dosed for 6–9 days with a panel of 47 drugs [27]. Sensitivity for drug toxicity detection was 65% for iPSC-HH-MPCCs and 70% for PHH-MPCCs for the chosen drug set, while it was 35% for SCHHs. Permission was obtained from Nature Publishing group to reproduce panels (a), (c), and (e). Permission was obtained from John Wiley and Sons to reproduce panels (d) and (f).

Microfabrication tools adapted from the semiconductor industry allow for the creation of heterogeneous surfaces with precise features that can range in sizes from a few nanometers to micrometers depending on the type of tools utilized [16]. In the case of the liver, Khetani and Bhatia pioneered micropatterned cocultures (MPCCs) of primary human hepatocytes (PHHs) and stromal cells such that controlled cell-cell interactions (i.e., architecture) led to high and stable liver functions for 4-6 weeks* in vitro* as compared to unstable randomly distributed cocultures of the same two cell types (Figure 1) [14]. Soft-lithography that utilizes elastomeric polymers such as polydimethylsiloxane (PDMS) was used to miniaturize MPCCs in 24- and 96-well plate-based screening formats. Interestingly, a nonliver murine embryonic 3T3 fibroblast subclone (3T3-J2) induces higher functions in PHHs than even liver-derived stromal cells (manuscript in preparation), potentially due to the embryonic fibroblasts invoking developmental pathways that are complementary across the two species [15]. Nonetheless, MPCCs have been designed to be modular in that the stromal population can be modified without significantly affecting the hepatocyte homotypic interactions on the micropatterned domains, which are important for maintaining cell polarity. For instance, preestablished MPCCs were augmented with primary human Kupffer macrophages once the hepatic phenotype was stable after ~1 week in culture [17]. Stimulating the macrophages with bacterial-derived endotoxin, lipopolysaccharide (LPS), led to cytokine-mediated downregulation of specific cytochrome P450 (CYP450) enzymes in hepatocytes, which can affect DILI outcomes. We are now augmenting MPCCs with liver sinusoidal endothelial cells and hepatic stellate cells to enable the crosstalk between liver cell types in modeling different types of DILI.

MPCCs created with either primary human or rat hepatocytes were incubated for 5 to 9 days with a set of 35 drugs with known DILI liabilities in the clinic, while 10 drugs served as non-liver-toxic controls [18]. The doses tested ranged from 1*∗C*_max⁡_ to 100*∗C*_max⁡_ for each drug, where *C*_max⁡_ is the reported maximal drug concentration in human plasma. Xu et al. justified the use of doses up to 100*∗C*_max⁡_ due to interindividual differences in drug concentrations within the liver [12]. The DILI detection results using MPCCs proved several key hypotheses. First, repeated drug dosing improved sensitivity for DILI detection without compromising specificity (i.e., no additional false positives compared to those with shorter durations of drug dosing in sandwich cultures). Second, secreted biomarkers such as albumin and urea were as sensitive for DILI detection as the more classical toxicity marker, ATP, which allows monitoring of the same well over time with repeated drug dosing and conserves the use of expensive and limited PHHs. Third, human MPCCs were more sensitive (65.7%) than their rat MPCC counterparts (48.6%) for human DILI detection. For an additional 19 drugs with the highest DILI concern as classified by the Food and Drug Administration (FDA) [19], human MPCCs displayed a sensitivity of 100% when at least 2 PHH donors were used for testing. Overall, human MPCCs improved the sensitivity by 2.3-fold compared to ECM-sandwich cultures (~28.6%) created using the same PHH donor and dosed with the same set of drugs.

**Figure 2:**
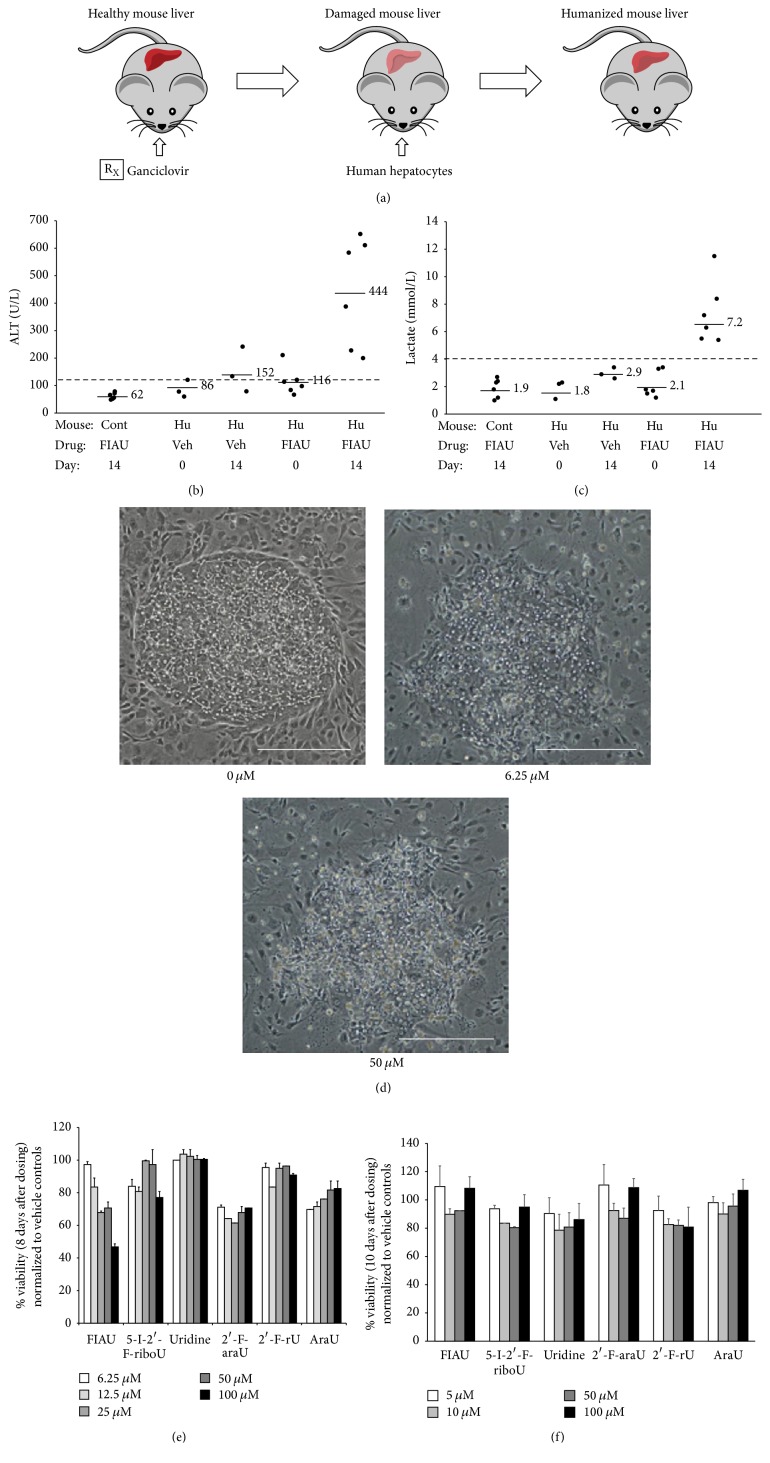
Fialuridine toxicity assessment in humanized rodents and micropatterned cocultures (MPCCs) containing either primary human hepatocytes (PHH) or primary rat hepatocytes. Mice were briefly exposed to a nontoxic dose of ganciclovir to ablate murine liver cells (panel (a)) [118]. PHHs were transplanted into 8-week-old mice and the humanized liver was established for 8 weeks prior to toxicology studies. Humanized and control nonhumanized mice were dosed with vehicle (0.5% dimethylsulfoxide) or 2.5 mg/kg/d fialuridine for 14 days by oral gavage. Plasma alanine aminotransferase or ALT (panel (b)) and lactate (panel (c)) levels were measured on days 0 and 14 [78]. Each dot in the graphs of panels (b) and (c) represents 1 mouse, the solid lines adjacent to the dots represent averages for each sample group, and the dashed line across each graph represents the upper limit of normal. PHH-MPCCs were dosed for 8 days with 0, 6.25, or 50 *µ*M fialuridine and deteriorating hepatocyte morphology was recorded with increasing dose (panel (d)) [20]. Scale bars on images represent ~250 *µ*m. In addition to fialuridine (5-I-2′-F-araU), PHH-MPCCs were dosed for 8 days with several doses of 5 other analog compounds. Mitochondrial activity was assessed using the MTT assay and normalized to vehicle only controls (panel (e)). Only fialuridine caused a dose-dependent toxicity in PHH-MPCCs. On the other hand, no dose-dependent toxicity was observed in MPCCs created using primary rat hepatocytes and dosed with the compounds for 8 days (panel (f)).

In an interesting case study, MPCCs, but not sandwich cultures of PHHs, have been shown to detect the toxicity of fialuridine [20], a nucleoside analog drug for hepatitis B viral infection that caused the deaths of 5 patients in clinical trials due to lactic acidosis [21]. Such a drastic human DILI outcome was not predicted previously by studies in rats, dogs, or monkeys. As in the clinic, dosing human MPCCs with fialuridine for 4–21 days led to dose- and time-dependent toxicity as assessed by several endpoints such as mitochondrial activity, albumin, urea, and morphological alterations (Figure 2). The toxicity of fialuridine could be compared against its structural analogs towards enabling a structure-activity relationship (SAR) approach. On the other hand, rat MPCCs did not display the same extent of fialuridine toxicity as their human counterparts even after 28 days of dosing. Interestingly, urea secretion and CYP3A activity decreased in rat MPCCs with long-term fialuridine dosing, which is in agreement with a previous* in vivo *study [22]. Thus, MPCCs created using hepatocytes from different types of animals (i.e., mouse, rat, dog, and monkey) and humans can serve to elucidate key differences in species-specific DILI progression and thus allow for selection of the most appropriate species for FDA-required* in vivo* animal investigations.

Other groups have created different variations of micropatterned cocultures containing hepatocytes and stromal cells. For instance, Zinchenko et al. used photo- and soft-lithographic techniques to create micropatterned cocultures of primary rat hepatocytes and Kupffer macrophages [23]; however, this configuration displayed a decline in hepatic functions over 10 days, whereas the use of 3T3-J2 fibroblasts leads to stable functions for at least 4 weeks in both primary rat [15] and human hepatocyte cultures [14]. Cho et al. used 3T3-J2s as the stromal cell type but utilized PDMS stencils to culture rat hepatocytes on top of micropatterned fibroblast colonies [24]. The authors found higher functions in this “layered” configuration as compared to the configuration in which both cell types were in the same plane of culture. In a configuration containing three cell types, Liu et al. micropatterned electrospun fibrous scaffolds to in turn create micropatterns of rat hepatocytes, NIH-3T3 murine embryonic fibroblasts, and human umbilical vein endothelial cells [25]. Such tricultures led to the formation of hepatic spheroids that secreted albumin and urea as well as displayed CYP450 activities for 15 days. These tricultures were shown to be useful for prediction of drug clearance and for drug-mediated modulation of CYP450 activities; however, demonstration of their utility for drug toxicity detection is pending.

PHHs are considered the gold standard for constructing human liver models but are limited in the genetic diversity available to understand interindividual differences in DILI outcomes. On the other hand, induced pluripotent stem cells (iPSCs) can be derived from many patients, including those who have known susceptibilities to toxicity due to certain drugs. Furthermore, unlike PHHs, iPSCs are a renewable cell source for sustainable drug screening using the same set of donors. Protocols to differentiate iPSCs down the hepatic lineage use growth factors inspired from* in vivo* liver development as well as small molecules; however, adult liver functions remain low (<10%) when relying on such protocols alone [26]. We have shown that the MPCC platform is also useful to further differentiate and stabilize functions of iPSC-derived human hepatocyte-like cells (iPSC-HHs) for at least 4 weeks* in vitro *(Figure 1) [27]. Even though the iPSC-HHs in MPCCs were still not as differentiated as PHHs cultured in the same system, we were nonetheless able to demonstrate iPSC-HH utility for DILI detection. In particular, iPSC-HH-based MPCCs dosed with a set of 47 drugs for 6 days yielded a sensitivity (65%) that was remarkably similar to sensitivity in PHH-based MPCCs (70%) dosed with the same drugs, while specificity in both models was 100% with a set of 10 non-liver-toxic drugs. These results suggest that iPSC-HH-based MPCCs may be ready for an initial drug toxicity screen during drug development; however, mechanistic inquiries into DILI outcomes will require further probing of active pathways within iPSC-HHs relative to PHHs.

## 4. Spheroidal and Bioprinted Cultures

Hepatocyte spheroids have shown improved functions over conventional 2D pure monolayers for drug screening applications, likely due to the establishment of homotypic cell-cell interactions and the presence of ECM within and around the spheroids [6]. Cell lines, primary hepatocytes, and iPSC-HHs cultured in spheroids have all shown utility for drug toxicity screening [28–33]. Additionally, PHH spheroids have been shown to replicate certain liver pathologies such as steatosis and cholestasis, allowing for the assessment of DILI in a diseased background [34].

Engineered scaffolds and channels can aid in the assembly of spheroids that are more consistent in size than is possible with random configurations. For instance, Kostadinova et al. first deposited a mixture of liver stromal cells onto a porous nylon scaffold, followed by seeding of PHHs [31]. Secretion of albumin, transferrin, and fibrinogen was maintained for ~77 days. This model detected clinically relevant drug toxicity, including species-specific drug effects with higher sensitivity than monolayer cultures. However, the gene expression profiles of this complex coculture changed over time, suggesting that it is not trivial to control the growth and interactions of the various cell types in a randomly distributed configuration. In another study, Tong et al. immobilized hepatocyte spheroids between a glass coverslip and an ultrathin porous Parylene C membrane that were both surface-modified with polyethylene glycol (PEG) and galactose for enhanced spheroid formation and maintenance [35]. In such a “constrained spheroid” configuration, loss of spheroids over time due to medium changes and/or perfusion was minimized.

**Figure 3:**
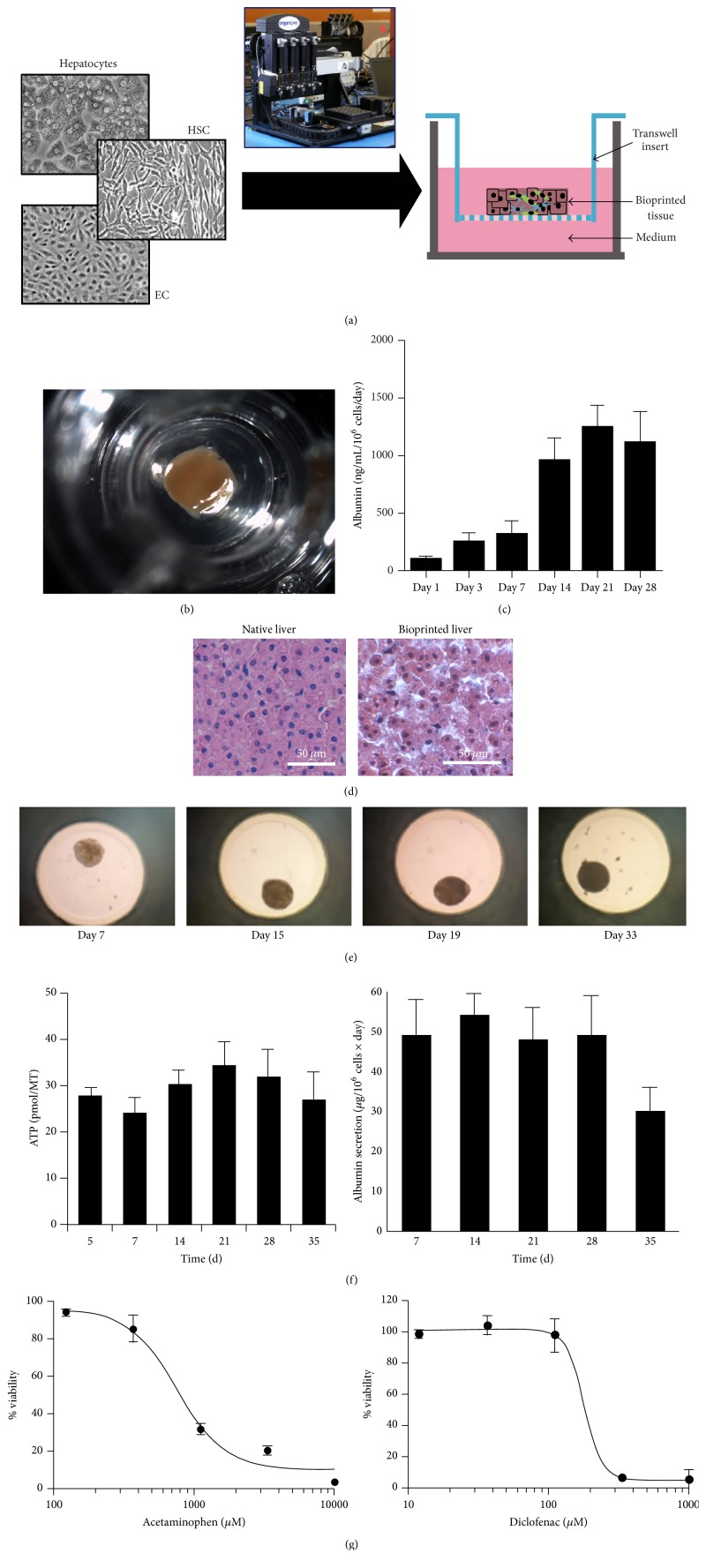
Spheroidal cocultures containing primary human hepatocytes (PHHs). (a) Schematic of transverse cross section of bioprinted liver tissue from Organovo containing PHHs, endothelial cells (ECs), and hepatic stellate cells (HSCs). Image of the 3D bioprinting instrument is shown as well. (b) Gross image of bioprinted human liver tissue with 2.5 mm diameter and 0.5 mm thickness. (c) Albumin secretion in bioprinted liver tissues over time. (d) Comparison of H&E stained native liver and bioprinted liver. Images and data for panels (a)–(d) were provided by Organovo, Inc. (e) Human liver spheroids from InSphero contain PHHs, ECs, and Kupffer macrophages and can maintain their size for at least 33 days* in vitro* [36]. MT indicates individual micro tissue. (f) Human liver spheroids maintain intracellular ATP content and secrete albumin for 35 days. (g) Utility of InSphero human liver spheroids for measuring dose-dependent toxicity of different drugs following an incubation period of 14 days.

A specialized plate has been developed for creating hanging drops of mixed liver cells such as PHHs, endothelial cells, and Kupffer macrophages that can lead to the formation of spheroids of controlled diameters (i.e., 253 ± 7.4 *μ*m). These spheroids are then transferred to another multiwell plate for drug testing [36] (Figure 3). Such spheroids remain viable and secrete albumin for ~1 month. Dose-dependent toxicity of acetaminophen, diclofenac, and trovafloxacin has been observed in this platform. Trovafloxacin toxicity was further sensitive to activation of Kupffer macrophages via LPS. This system has been adapted to both hepatic cell lines (i.e., HepG2) and PHHs. In another engineered platform, Miyamoto et al. utilized a Tapered Stencil for Cluster Culture (TASCL) device to form HepG2 spheroids [37].

Culture of iPSC-HHs in spheroidal configurations, such as in collagen matrices, can also improve their functions relative to monolayer controls [32]. Takayama et al. utilized spheroidal cultures of iPSC-HHs created using a “nanopillar plate” to assess the toxicity of 24 drugs [38]. iPSC-HHs and HepG2 spheroids were exposed to the toxic drugs for 24 hours and cell viability was assessed using a WST-8 assay. iPSC-HH spheroids were found to be more sensitive to the toxins as compared to the HepG2 spheroids; however, the sensitivity of iPSC-HHs to the toxins was lower than that observed with plated primary hepatocyte monolayers.

Bioprinting can also be utilized to create spheroidal structures by positioning liver stromal cells (i.e., stellate cells, endothelial cells) relative to hepatocytes, which can lead to a compartmentalized architecture and microvascular networks (Figure 3). These scaffold-free “organoids” were shown to detect the toxicity of a drug that had been deemed safe in animal studies but caused human DILI (http://www.organovo.com/). Ma et al. utilized rapid, digital 3D bioprinting to print iPSC-HHs, endothelial cells, and adipose-derived stem cells in a microscale hexagonal architecture embedded in hydrogel that mimics the liver lobule architecture [39]. Whether such a complex architecture will lead to higher sensitivity for DILI detection than cells randomly distributed in a spheroidal structure has not yet been determined.

Naturally derived (i.e., alginate, chitosan, and cellulose) and synthetic biomaterials (i.e., PEG) can be utilized for embedding aggregated hepatocytes [6]. Use of biomaterials allows spatiotemporal tuning of mechanical and biochemical properties that the cells experience. For instance, the previously described MPCC platform can be first used to control cell-cell interactions between PHHs and stromal cells. Then, the entire micropatterned monolayer can be detached using collagenase treatment and embedded in PEG hydrogels that not only are biocompatible, but also provide control over mechanical properties via customization of chain length and biochemical properties by tethering active ligands such as growth factors [40, 41]. Micropatterned PHH/stromal cell clusters encapsulated in PEG displayed higher liver functions than encapsulated random cocultures. A microfluidic droplet generator can also be used to generate PEG-based hepatic microtissues, which are more amenable to high-throughput drug studies than bulk gels. In a study utilizing a naturally derived biomaterial, Tasnim et al. encapsulated human pluripotent stem cell-derived hepatocyte-like cells (hPSC-HLCs) in galactosylated cellulosic sponges [42]. The sponges promoted spheroid formation and the porous network served as a physical constraint to maintain spheroid sizes. The spheroid cultures were dosed with acetaminophen, troglitazone, and methotrexate and compared to conventional 2D cultures of both hPSC-HLCs and PHHs. hPSC-HLC spheroids were more sensitive to the toxins than the hPSC-HLC conventional cultures, and spheroid responses to the toxins were similar to that of PHHs.

## 5. Perfusion Systems

Even though hepatocytes in the liver are protected from flow-induced shear stress by the endothelial fenestrae, flow can cause gradients of oxygen, nutrients, and hormones, which have been shown to lead to zonation or differential functions in hepatocytes across the length of the sinusoid [43]. DILI can thus manifest itself with a zonal pattern dependent on the mechanism of action of the drug and its metabolism by specific isoenzymes in the hepatocytes. A parallel-plate bioreactor with oxygen gradients has been used to induce a zonal pattern of CYP450s in rat hepatocytes, which led to a zonal pattern in acetaminophen toxicity, particularly in low oxygen regions where CYP450 enzymes were expressed at higher levels than expression in high oxygen regions [44, 45].

In addition to inducing zonal hepatic functions, several investigators have postulated that flow can allow better nutrient exchange and removal of waste products, which can lead to higher hepatic functions than static cultures. Novik et al. observed production of drug metabolites at greater rates in PHH/endothelial cell cocultures subjected to flow as compared to static cocultures [46]. Sivaraman et al. perfused preformed hepatic aggregates adhered to the collagen-coated walls of an array of microchannels and observed higher hepatic functions than static collagen sandwich cultures [47]. Esch et al. subjected multicellular cocultures of PHHs and stromal cells (fibroblasts, stellate cells, and Kupffer macrophages) to perfusion and found higher albumin and urea secretion than in static controls [48]. Instead of subjecting hepatocytes to shear stress via direct perfusion, Lee et al. subjected hepatic aggregates to nutrient exchange via flow in an adjacent channel that had through-holes similar to the fenestrae of the endothelial layer in the liver [49]. Perfusion in the channels was gravity-driven with the inlet and outlet reservoirs containing different volumes of culture media, which simplified the device by eliminating the need for external pumps. Other investigators are also incorporating gravity-driven flow into their liver devices [48, 50].

**Figure 4:**
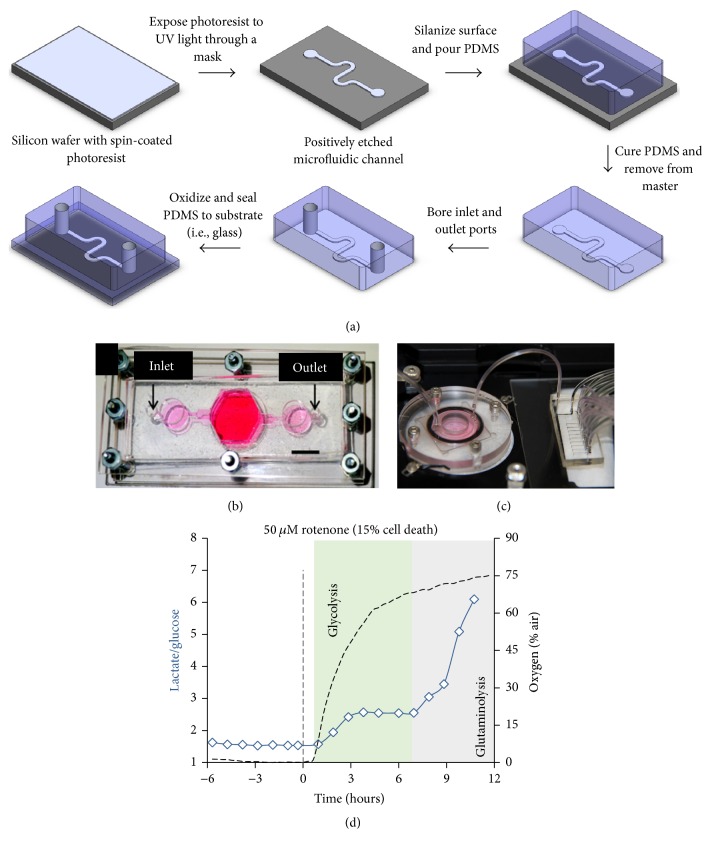
Liver-on-a-chip devices. (a) Soft-lithographic process utilizing photoresist-coated silicon wafers and molding of polydimethylsiloxane (PDMS) on the wafers to create microfluidic devices with channels for cell seeding and inlet/outlet ports for culture medium perfusion. (b) Top-view of an assembled bioreactor with inlet and outlet fluidic ports [54]. (c) Photo of a bioreactor hooked up to a switchboard that can be configured for real-time measurement of metabolites [55]. (d) Measurement of glucose uptake and lactate production in the device of panel (c) following dosing with rotenone. Permission was obtained from IOP Publishing to reproduce panel (b).

In addition to perfusion of culture medium, microfluidic devices can also be utilized to control the spatial arrangement of cells to yield the type of architecture (i.e., control over cell-cell interactions) that has been shown to improve liver functions (Figure 4). For instance, Kobayashi et al. utilized microfluidic and micronozzle devices to coculture HepG2 and Swiss 3T3 fibroblasts in a stripe-patterned hydrogel sheet, which allowed for the control of the direction of proliferation and the formation of arrays of rod-like organoids inside the hydrogel [51]. Skardal et al. mixed HepG2 cells with a hydrogel designed to mimic ECM prior to introducing the mixture into the parallel channels of a microfluidic device [52]. The cells were then exposed to ethyl alcohol and cell damage was assessed. Ma et al. also used a microfluidics-based biomimetic method to fabricate a 3D liver lobule-like microtissue [53]. The microtissue consisted of HepG2 cells and an immortal human aortic endothelial cell line to mimic the presence of liver endothelial cells and was able to metabolize acetaminophen, isoniazid, and rifampicin, and toxicity was assessed via fluorescein diacetate/propidium iodide costaining. Bhise et al. encapsulated HepG2/C3A spheroids in a photo-cross-linkable gelatin methacryloyl hydrogel and printed droplets in the cell culture chamber of a microfluidic bioreactor [54]. The encapsulated and perfused spheroids functioned for ~30 days as assessed by several markers, such as secretion of albumin and alpha-1-antitrypsin, and immunostaining for the tight junction protein, zona occludens-1. Furthermore, a 15 mM dose of acetaminophen induced a toxic response in the spheroids as expected from* in vivo *rat studies.

Microfluidic devices are inherently low-throughput for testing a large panel of drugs and are more difficult to set up and handle relative to industry-standard multiwell plates. Therefore, incorporation of real-time monitoring of toxicity biomarkers in microfluidic devices can not only aid in ease of usability, but also provide more rapid assessment of drug effects than is possible with conventional assays. For instance, Bavli et al. assessed mitochondrial function and glucose metabolism in real time on a liver-on-a-chip device where 3D aggregates of HepG2/C3A cells were exposed to rotenone and troglitazone for 24 hours [55]. Oxygen uptake dropped within a few minutes following drug exposure while the metabolic shift from oxidative phosphorylation to glycolysis occurred 3–6 hours later, coupled with a gradual change in glucose and lactate fluxes. Rennert et al. established a liver organoid consisting of human umbilical vein endothelial cells and monocyte-derived macrophages in the vascular plane and HepaRG and LX-2 stellate cells (immortalized line) in the hepatic plane with a membrane mimicking the space of Disse in a microfluidic perfused biochip [56]. Luminescent-based sensor spots were integrated in the chip to allow for real-time measurement of oxygen consumption levels. Finally, Vernetti et al. created a platform in which PHHs, EA.hy926 endothelial cells, U937 monocytes, and LX-2 stellate cells were sequentially layered in a microfluidic device that was continuously perfused and had fluorescent protein biosensors inside select PHHs [57]. Troglitazone and nimesulide toxicity were assessed whereas caffeine was used as a negative control. Increased toxicity of trovafloxacin was observed when cultures were costimulated with LPS. The model also demonstrated increased stellate cell migration and expression of alpha-smooth muscle actin and collagen in response to methotrexate, indicating fibrotic activation. The aforementioned multicellular culture models show the latest trends in liver-on-a-chip devices, which are being designed to include as many of the liver cell types as possible to allow for the crosstalk necessary (via paracrine signaling, ECM deposition, and cell-cell contact) to elicit more complex DILI outcomes than possible with PHH-only culture platforms.

Microfluidic perfusion is also useful to create organs-on-a-chip platforms in which the liver compartment is linked to compartments containing cells of other tissue types towards measuring how drug metabolism by liver cells affects other cell types [5]. Viravaidya et al. created one of the earlier organs-on-a-chip models in which cell lines were used to model lung, liver, and fat compartments that were linked with microfluidic flow to investigate the biodistribution of compounds [58]. Chouca-Snouber et al. created a microfluidic biochip that modeled the liver compartment with HepG2/C3a or HepaRG cell lines and the kidney compartment with the MDCK cell line [59]. The synergistic reaction between the two tissue types was demonstrated via ifosfamide dosing. HepaRG cells, but not HepG2/C3a, metabolized ifosfamide into a nephrotoxic metabolite. Materne et al. created a biochip consisting of HepaRG and primary human hepatic stellate cell spheroids and differentiated NT2 cell neurospheres [60]. After 2 weeks of repeated dosing with the neurotoxin, 2,5-hexanedione, the cocultures were more sensitive than the single-tissue cultures in the biochip. Esch et al. connected HepG2/C3a cells in a liver compartment on a biochip with an intestinal compartment containing Caco-2 (absorptive) and HT29-MTX (mucus-secreting) cells to investigate nanoparticle toxicity [61]. When both the liver and intestinal chambers were exposed to polystyrene nanoparticles, increased cellular damage was observed as compared to liver-only exposure. Other groups have used biochips to study liver-skin interactions in troglitazone-induced toxicity [62] and topical substance exposure [63]. Sung et al. created a microfluidic device that utilized 3D cultures of colon and liver cell lines to evaluate the toxic effects of anticancer drugs [64]. The results showed that, as compared to static 96-well cultures, the microfluidic device was able to more accurately reproduce liver metabolism of the cancer drug, tegafur, to its metabolite, 5-fluorouracil, which caused expected toxicity to the cancer compartment. Finally, several groups have created four organ-chips for assessing multiorgan toxicity. Maschmeyer et al. combined intestine, skin, liver, and kidney modules onto a chip and assessed viability and functionality of the cells although no drug toxicity studies were carried out [65]. Oleaga et al. combined cardiac, muscle, neuronal, and liver modules and looked at doxorubicin, atorvastatin, valproic acid, acetaminophen, and N-acetyl-m-aminophenol toxicities [66].

## 6. Liver Slices

In contrast to many cell-based models, precision cut liver slices (PCLS) retain tissue architecture and contain all cell types of the liver [67]. However, PCLS display a rapid decline in hepatic functionality when placed in a static culture medium [68]. Microfluidic devices have been used to extend the functional lifetime of PCLS. For instance, rat PCLS were embedded in Matrigel and placed in a microfluidic device to allow production of phase I drug metabolites after 72 hours of culture (~10% of metabolites produced relative to the day of isolation) [69]. van Midwoud et al. cultured human PCLS in a microfluidic device that was coupled with high-performance liquid chromatography (HPLC) for the detection of unstable metabolites [70]. While microfluidic perfusion can improve their longevity [71, 72], PCLS still do not display high levels of functions for more than a few days, which severely limits chronic drug dosing studies in which a stable liver phenotype is required for at least multiple weeks. Nonetheless, PCLS provide for the most intact* in vitro *human liver model for testing specific hypotheses in a 24–72-hour timeframe.

## 7. Humanized Rodent Models

In certain types of rodent models, the liver can be repopulated with PHHs such that human-specific drug metabolites and DILI can be measured in an* in vivo* context [73, 74]. In some instances, the repopulated humanized livers can express phase I and II metabolism enzymes and some transporters at* in vivo*-like levels [75, 76]. Several groups have subsequently used humanized rodent liver models to study mechanisms of drug toxicity. For instance, Yamada et al. used a humanized rodent model to supplement* in vitro *human hepatocyte data to assess the mode of action by which sodium phenobarbital produces tumors in rodents and the relevance of this data for human risk assessment [77]. Xu et al. showed that although control TK-NOG mice tolerated high doses of fialuridine, humanized TK-NOG mice showed dose-dependent toxicity by 3 days [78]. In particular, humanized liver regions showed vacuolar changes, the presence of enlarged fat vacuoles, and mitochondrial changes, which were present in human subjects (Figure 2). Additionally, the same group demonstrated dose-dependent toxicity of bosentan in humanized mice relative to the control mice [79]. Serum ALT (alanine aminotransferase) levels, bile acid accumulation, and the analyses of other liver plasma injury markers in humanized mice were consistent with findings in bosentan-treated human subjects.

Humanized rodent models in which only PHHs are used cannot recapitulate the types of human DILI where the adaptive immune system plays an important role. For example, although Kakuni et al. were able to recapitulate troglitazone toxicity in a chimeric mouse with a humanized liver, immune-mediated reactions associated with troglitazone toxicity could not be studied due to the use of an immunodeficient SCID mouse [80]. Therefore, more recent humanized rodent models have incorporated both human liver and human immune cells, though their utility for studying DILI is pending [81–83]. However, there remains room for improvement to better mimic the human immune system in these rodents.

Some groups have implanted PHHs in ectopic sites instead of directly transplanting them into a compromised rodent liver. For instance, Chen et al. first cultured PHHs and supportive stromal cells (3T3-J2 fibroblasts, immortalized endothelial cells) in PEG hydrogels before implanting these constructs in the subcutaneous or intraperitoneal regions of immune-competent mice [84]. The human constructs survived for up to 3 months in the intraperitoneal region. Finally, Ohashi et al. transplanted PHHs embedded in Matrigel in the kidney capsule with administration of an agonistic antibody against c-Met (also called hepatocyte growth factor receptor) to stabilize the PHHs for 3–6 months* in vivo* [85].

## 8. High Content Readouts

High content screening (HCS) of multiplexed fluorescent readouts can be used to obtain an understanding of mechanisms underlying DILI at the organelle level. HCS systems (i.e., Thermo-Fisher ArrayScan, Molecular Devices ImageXpress, GE Healthcare IN Cell Analyzer) couple automated and multispectral epifluorescent microscopy with software for real-time analysis of fluorescent intensities within individual cells. Here, we will focus on HCS studies in which toxicity tests were performed on human-relevant cells and will refer the reader to several other reviews that cover HCS technologies in greater detail [86–88].

HCS for DILI detection was initially implemented by O'Brien et al. using HepG2 cells [89] and later extended by Xu et al. to short-term ECM-sandwich cultures of PHHs [12]. Garside et al. then used HCS in a 384-well plate format to investigate the effects of 144 drugs on HepG2 cells cultured in the absence or presence of rat S9 fraction (for generating drug metabolites) and on cryopreserved PHHs [90]. The parameters assessed captured several mechanisms of DILI including cell number, reactive oxygen species, mitochondrial membrane potential, apoptosis, cell cycle arrest, cell stress response, phospholipidosis, and neutral lipid accumulation. HCS has also been applied to cocultures of PHHs and stromal cells by developing computational algorithms that can separate out the fluorescent intensities from multiple cell types based on nuclear size/shape or other cell type-specific signals [91]. The “Integrated Discrete Multiple Organ Coculture” (IdMOC) system was used to coculture PHHs and 3T3-L1 cells in separate wells that share culture media for the assessment of multiple endpoints after 4-aminophenol and cyclophosphamide exposure [92]. HCS has recently been adapted to monolayers of iPSC-HHs [93, 94]. Pradip et al. used HCS in human iPSC-HHs to evaluate drugs known to cause hepatotoxicity through steatosis and phospholipidosis and benchmarked them to the HepG2 cell line [95]. While the aforementioned HCS studies provide information on the effects of drugs on various endpoints, the overall sensitivity (~50–58%) is typically not improved significantly over non-HCS based assays (i.e., albumin, urea, and ATP), potentially due to the lack of other processes in such screens (i.e., transporters, interaction of hepatocytes with activated liver stromal cells, and innate and adaptive immune responses) that are relevant for the progression of DILI in the clinic. Nonetheless, the ability to probe DILI mechanisms using multiple endpoints is especially important when there is little to no information on the predicted or actual *C*_max⁡_ of a candidate compound in humans during the early stages of drug development.

Another type of high content readout involves toxicogenomics (TGx), which combines genomics (i.e., mRNA transcripts, microRNAs, DNA methylation patterns, and single nucleotide polymorphisms) and bioinformatics analyses to characterize genes and pathways underlying drugs' effects on cells [96]. Changes at the gene expression level may precede cellular damage and could thus be useful to identify the mechanisms by which a drug may cause injury following prolonged exposure. However, an FDA study showed that the human DILI potential of a drug can only be reasonably assessed using TGx analyses of* in vivo* studies in rats if the drug also produced significant elevation of ALT or TBL (total bilirubin) in the animal [97].

Human liver cultures can potentially complement TGx studies when no liver enzyme elevation is observed in animals. Rodrigues et al. exposed PHHs, HepaRG, HepG2, and human skin-derived precursor hepatic progenitor cells to acetaminophen [98]. Transcriptomics analysis showed comparable hepatotoxic effects among all the cell types except for HepG2, which did not show activation of liver damage. HepaRG was the most sensitive to liver damage, followed by human skin-derived precursor hepatic progenitor cells and PHHs. However, the culture method can lead to inherent gene expression changes in hepatocytes even in the absence of a drug stimulant. For instance, one study found significant gene expression changes when hepatocytes were cultured on collagen as opposed to Matrigel [99]. Ultimately, functionally stable engineered liver models may address such shortcomings so that TGx can be utilized during preclinical drug development for better prediction of clinical outcomes.

Gene expression can also be complemented with proteomics and metabolomics to allow for the study of DILI pathogenesis at multiple levels. We refer the readers to other reviews on the development and application of these tools for DILI detection [100, 101]. Here, it suffices to say that further validation using different drug sets across multiple laboratories with standardized data analysis schemes will be required before the aforementioned “-omics” technologies will be routinely employed in prospective drug development. Nonetheless, such tools provide a powerful means by which to study detailed molecular changes induced by drugs over time and we anticipate that their use with engineered human liver models will continue to grow.

## 9. *In Silico* Predictions

Quantitative structure-activity relationships (QSAR) can be used to determine whether any property of the chemical (i.e., structure) is an indicator of its potential to cause drug toxicity. There are multiple computational systems currently in use, some of which assess liver-specific toxicity [102]. For instance, Zhu et al. used 289 compounds to create* in silico* models based on chemical descriptors and* in vitro *toxicity endpoints and found that utilizing both the descriptors and the endpoints resulted in better toxicity prediction as compared to using the chemical descriptors alone [103]. Mulliner et al. have recently compiled a large set of* in vivo *hepatotoxicity data and used a machine learning approach to create models that are useful for the* in silico* safety assessment of new molecular entities during the early stages of drug development [104]. Another QSAR model that has recently been updated is the OpenVirtualToxLab, which has moved away from using “training sets” [105]. In this way, any biases that come from specific training sets are removed. The ToxCast project by the US Environmental Protection Agency (EPA) has assessed several different types of* in vitro* assays that provide information on diverse molecular pathways that are modulated upon dosing with industrial chemicals and reference pharmaceuticals [106].

Some groups are creating computational models that quantitatively integrate mechanistic pathways implicated in DILI. For instance, DILIsym software simulates pathways and progression of endpoints pertinent in DILI [107]. This software, when coupled with* in vitro *data, can model some species-specific aspects of mitochondrial effects, bile acid toxicity, and innate immune responses [108]. The “Virtual Liver” software by Strand Life Sciences can, in conjunction with* in vitro* assays, provide mechanistic insights into how a drug impacts known DILI pathways [109]. Finally, while retrospective validation of novel culture platforms for DILI prediction has traditionally used *C*_max⁡_ values of drugs that have gone through human clinical trials, in a prospective drug screening campaign, physiologically based pharmacokinetic (PBPK) modeling can be useful to extrapolate between* in vitro* and* in vivo* exposure conditions [110, 111]. Such extrapolation can help establish a margin of safety (i.e., therapeutic index) for candidate compounds when comparing the concentration range that can cause toxicity relative to the concentration range that can bind to the molecular target of interest for potential efficacious effects.

## 10. Conclusions and Future Outlook

Human DILI is a major global health burden and it has become clear over many drug failures that animal studies are not sufficient to fully predict and understand human-relevant outcomes [3]. Furthermore, the idiosyncrasy of DILI in the clinic makes preclinical prediction even more challenging [1]. While the development of human liver models was initiated many decades ago with the isolation and culture of PHHs on ECM, the rapid functional decline of these cells outside of their native liver microenvironment limits the prediction of clinical DILI outcomes [12, 14]. Over the last few years, engineers have developed tools that now allow for more precise control over the microenvironment of PHHs such that functions can be stabilized for several weeks to months (Table 1). Initially, rat hepatocytes and cancerous/immortalized hepatic cell lines were used to test the utility of such tools, but now translation to PHHs is progressing rapidly with the realization that these cells are the closest representation of the human liver. Additionally, human liver models are being coupled with models of other organs/tissues to better predict and understand how drug metabolism in the liver affects toxicity in other tissue types. Such integration is being done both* in vitro* using microfluidic perfusion and* in vivo* with humanized rodent livers.

Over many years of research, the field of engineered liver models has come to realize some important considerations in the design of such models. First, PHHs can be functionally stabilized for many weeks even when the culture model does not always mimic the exact architecture or composition of the liver (i.e., disorganized 3D spheroids, cocultures with murine embryonic fibroblasts) [112]. Second, exercising control over cell-cell interactions, both homotypic and heterotypic with stromal cells in either monolayers or bioprinted tissues, is important to enhance PHH functions reproducibly across many experiments/donors and prevent premature decline. Third, inclusion of multiple liver cell types at physiologic ratios* in vitro *can be useful for modeling certain types of DILI where heterotypic cell-cell communication between two or more liver cell types is important. For instance, activation of Kupffer macrophages into a more inflamed state can downregulate certain CYP450s in PHHs, which can modulate the toxicity of drugs that are metabolized by those enzymes [17]. Additionally, drugs can activate hepatic stellate cells into becoming myofibroblasts that deposit excessive amounts of ECM and secrete cytokines which affect hepatocyte functions due to the changing microenvironment [57]. It is not yet clear how to incorporate biliary epithelial cells in liver models such that they interface with the bile canaliculi between hepatocytes and drain the canalicular contents. Such a directional flow would be important to properly determine drug disposition and toxicity to other organs.

The aforementioned technological developments have already and will continue to improve the sensitivity of human DILI detection and provide insights into the mechanisms of different types of DILI. However, with several models now available in the marketplace, selection criteria need to be applied to select appropriate models for specific phases of the drug development pipeline. In our view, the choice of the culture model is dependent on the hypotheses being tested and the confidence that the chosen culture model has the levels of sensitivity and specificity that are acceptable for the type of throughput desired. For example, renewable iPSC-HHs or even hepatic cell lines (i.e., HepaRG) when cultured in engineered platforms to improve their differentiated functions can be used to identify highly toxic compounds very early in the drug development pipeline with good specificity (i.e., low false positives) [27]. These compounds can then be subjected to medicinal chemistry to reduce or eliminate the severe toxicity with an appropriate safety margin. In the absence of *C*_max⁡_ information for a compound, it is important to determine a safety margin using* in vitro* toxicity data and binding affinity of the drug to the molecular target of interest. PBPK modeling can also aid in extrapolating critical information on pharmacokinetic parameters that could be potentially important* in vivo* [110, 111]. In later stages of drug development, micropatterned cocultures (containing PHHs and liver stromal cells) and/or 3D aggregates of controlled sizes (created using engineered scaffolds or bioprinted) in multiwell plates can be used to further probe the toxic effects of lead candidate compounds following chronic dosing [18]. As a lead candidate progresses through the pipeline, organs-on-a-chip platforms could be used to determine how different tissue types interact to produce toxicity in one or more tissue types [113]. There is always a chance that a candidate drug is flagged as toxic only when the most complex/complete culture system such as an organ-on-a-chip is utilized; however, if a low-throughput but high content model like organs-on-a-chip were utilized in the early stages of drug development, it may create bottlenecks in testing many drug analogs in multiple drug classes. Thus, the needs for throughput and cost have to be balanced with the sensitivity/specificity of the culture model being utilized. Even with the need for such a balance, the aforementioned iterative use of progressively more complex human liver models still provides a significantly faster and cheaper tiered testing strategy than afforded by the slow and sometimes entirely misleading animal testing. Certainly, the expectation is that testing on human-relevant models will reduce attrition in clinical trials, which constitute a major cost center in the $3–5B and 12–15 years that it now takes to bring a successful drug to the market [114, 115].

Even with considerable progress in the development of increasingly complex human liver platforms, some key questions/issues will need to be addressed moving forward. First, it will be important to rely on similar endpoints and data normalization schemes (i.e., based on cell number, protein, and/or RNA levels) when showing functionality and stability of a given culture system so that the data can be compared across different laboratories using the same system and across different types of engineered systems. Some markers, such as albumin and CYP3A4 activity, are commonly employed for appraising PHH functions, but the community will need to agree upon which PHH markers are appropriate for specific applications and how best to demonstrate the phenotype of other cell types in the liver. Second, it is important to compare gene expression and functions of liver cells in culture over time to fresh tissue and freshly isolated cell counterparts (prior to any plating) from the same donor(s) in order to determine the extent to which cultured cells deviate from the* in vivo*-like phenotype and which pathways are affected more than others. With the increased use of commercially available cryopreserved cells, fresh tissues/cells are not always available. In that case, we believe that, at the very least, the gene expression of cultured cells over time should be compared against cells immediately after thawing. Finally, whether 3D architecture in the form of spheroids and bioprinted constructs will yield greater advances for DILI prediction than engineered 2D models is not yet clear. We anticipate that consortia led by pharmaceutical companies, which are already evaluating different engineered systems against their drugs in-house, would be highly beneficial towards addressing this question. Ultimately,* in vitro *liver culture, whether 2D or 3D, is likely not going to mimic the* in vivo *liver phenotype perfectly but the degree to which it does will determine its utility for testing specific hypotheses in drug development and mechanistic inquiries into DILI.

It would be highly beneficial to reach a consensus as to which biomarkers to utilize to validate the utility of a platform for predicting different forms of DILI. Typically, endpoints such as ATP, albumin, urea, ALT, and lactate dehydrogenase (LDH) can be used to appraise the level of hepatic injury nondestructively in the same culture over time. Some combination of these endpoints used with stable PHH cultures can provide upwards of ~70% sensitivity for identification of drugs from several different classes as “toxic” [18]. HCS provides additional endpoints (i.e., mitochondrial membrane potential, reactive oxygen species, phospholipidosis, and lipid accumulation) to better elucidate mechanisms underlying DILI [12]. Furthermore, toxicogenomics, proteomics, and metabolomics can be used to provide indications of diverse molecular pathways that are affected by drug treatment even before overt cell injury. However, which of the aforementioned endpoints and analyses constitutes a “minimum essential set” for highly sensitive prediction of hepatotoxicity, especially during early drug development, is not yet clear. As more liver cell types are interrogated* in vitro*, we anticipate that consensus will also have to be reached on nonhepatic endpoints that are important for the prediction of those types of DILI in which heterotypic cell-cell communication plays an integral role.

Several studies utilizing cell lines, PHHs and iPSC-HHs, have shown that even some so-called “idiosyncratic” toxins (i.e., zafirlukast, troglitazone, diclofenac, and clozapine) can be detected using cellular stress markers [12, 89], potentially because such hepatic stress is a first step in the cascade of mechanisms that cause overt liver injury in specific patients with one or more covarying genetic (i.e., CYP450 polymorphisms) and environmental (i.e., coadministered drugs) factors. However, it is not currently possible to predict with* in vitro* approaches which* specific *individuals will go on to adapt to cell stress and which individuals will experience severe DILI. Creation of hundreds and even thousands of iPSC lines from different individuals, some with greater susceptibility to liver toxicity due to certain drugs, may ultimately be necessary to fully understand interindividual variations in DILI outcomes due to genetic makeup. Dosing iPSC-HH cultures with drugs under different diseased backgrounds (i.e., hepatitis B/C viral infection, steatosis, and inflammation) could also provide clues as to patient-specific DILI. However, iPSC-HH functions need to be further improved to be similar to PHHs before their potential for investigating DILI can be fully realized. Engineering tools have shown great promise in improving iPSC-HH functions, especially for DILI detection [27]; however, more progress needs to be made with not only further functional maturation, but also the use of standardized endpoints for appraising such maturity across different laboratories and culture systems.

In conclusion, different engineered human liver models can now be utilized in specific phases of drug development based on the posed hypotheses, throughput requirements, and budgetary constraints. Continued development and validation of such models will provide higher sensitivity in the prediction of different types of DILI and provide a better understanding of factors that can cause idiosyncratic DILI in certain patients. Ultimately, engineered human liver models will reduce the usage of animals in preclinical drug development and mitigate the risk of DILI to human patients in clinical trials and in the marketplace.

## Figures and Tables

**Table 1:** Models for assessing DILI.

Model	Benefits	Limitations	Example references
Conventional cultures/cocultures	(i) High-throughput (ii) Sandwich cultures can maintain cell polarity	(i) Usually lack liver stromal cells (ii) Loss of drug metabolizing capacity occurs within hours	[6, 10–13]

Micropatterned cocultures	(i) Controlled architecture allows for higher functions for 1-2 months (ii) Coculture allows for the study of drug toxicity in a diseased background (iii) Compatible with high content imaging readouts	(i) Usually lack all liver stromal cells (ii) Use nonhuman supporting cells	[14, 17, 18, 20, 27]

Spheroidal cultures/cocultures	(i) Multicellular interactions (ii) Maintenance of major liver functions for 1–3 months	(i) Difficult to control disorganized cell type interactions over time (ii) Necrosis in center of larger spheroids (iii) Size variability (iv) Incompatible with standard high content imaging equipment	[30–38]

Bioprinted cultures/cocultures	(i) Precise control of cell placement (ii) Multicellular interactions	(i) Printing resolution does not allow placement of individual cells (ii) Low-throughput (iii) Heterogeneous distribution of drugs across the bioprinted tissues	[39, 53, 54]

Perfused biochips	(i) Dynamic fluid flow for nutrient and waste exchange (ii) Sustained functionality for at least 1 month (iii) Can be combined with module for real-time toxicity endpoint readouts	(i) Binding of drugs to tubing (ii) Large dead volume requiring higher quantities of novel compounds (iii) Low-throughput (iv) Shear stress may cause lower hepatic functions (v) Can wash away built-up beneficial molecules	[44–52, 55–66]

Precision cut liver slices	(i) Retains native liver architecture and all liver cell types (ii) Can be combined with flow to improve functional lifetime	(i) Low-throughput (ii) Rapid decline in liver functions (iii) Heterogeneous distribution of drug within the slice	[67–72]

Humanized rodents	(i) Human-relevant toxicity profiles *in vivo* (ii) Can look at organ-organ interactions with a humanized liver background	(i) Variability in human hepatocyte engraftment efficiency (ii) Low-throughput (iii) Residual murine liver cells can cause confounding results (iv) Interactions with murine organs	[73–85]

## References

[1] Kaplowitz N. (2005). Idiosyncratic drug hepatotoxicity. *Nature Reviews Drug Discovery*.

[2] Abboud G., Kaplowitz N. (2007). Drug-induced liver injury. *Drug Safety*.

[3] Olson H., Betton G., Robinson D. (2000). Concordance of the toxicity of pharmaceuticals in humans and in animals. *Regulatory Toxicology and Pharmacology*.

[4] Guillouzo A. (1998). Liver cell models in in vitro toxicology. *Environmental Health Perspectives*.

[5] Yum K., Hong S. G., Healy K. E., Lee L. P. (2014). Physiologically relevant organs on chips. *Biotechnology Journal*.

[6] Godoy P., Hewitt N. J., Albrecht U. (2013). Recent advances in 2D and 3D in vitro systems using primary hepatocytes, alternative hepatocyte sources and non-parenchymal liver cells and their use in investigating mechanisms of hepatotoxicity, cell signaling and ADME. *Archives of Toxicology*.

[7] Donato M. T., Lahoz A., Castell J. V., Gómez-Lechón M. J. (2008). Cell lines: a tool for in vitro drug metabolism studies. *Current Drug Metabolism*.

[8] Anthérieu S., Rogue A., Fromenty B., Guillouzo A., Robin M.-A. (2011). Induction of vesicular steatosis by amiodarone and tetracycline is associated with up-regulation of lipogenic genes in heparg cells. *Hepatology*.

[9] Sharanek A., Burban A., Burbank M. (2016). Rho-kinase/myosin light chain kinase pathway plays a key role in the impairment of bile canaliculi dynamics induced by cholestatic drugs. *Scientific Reports*.

[10] Wilkening S., Stahl F., Bader A. (2003). Comparison of primary human hepatocytes and hepatoma cell line HepG2 with regard to their biotransformation properties. *Drug Metabolism and Disposition*.

[11] Gerets H. H. J., Tilmant K., Gerin B. (2012). Characterization of primary human hepatocytes, HepG2 cells, and HepaRG cells at the mRNA level and CYP activity in response to inducers and their predictivity for the detection of human hepatotoxins. *Cell Biology and Toxicology*.

[12] Xu J. J., Henstock P. V., Dunn M. C., Smith A. R., Chabot J. R., de Graaf D. (2008). Cellular imaging predictions of clinical drug-induced liver injury. *Toxicological Sciences*.

[13] Bi Y.-A., Kazolias D., Duignan D. B. (2006). Use of cryopreserved human hepatocytes in sandwich culture to measure hepatobiliary transport. *Drug Metabolism and Disposition*.

[14] Khetani S. R., Bhatia S. N. (2008). Microscale culture of human liver cells for drug development. *Nature Biotechnology*.

[15] Bhatia S. N., Balis U. J., Yarmush M. L., Toner M. (1999). Effect of cell-cell interactions in preservation of cellular phenotype: cocultivation of hepatocytes and nonparenchymal cells. *The FASEB Journal*.

[16] Qin D., Xia Y., Whitesides G. M. (2010). Soft lithography for micro- and nanoscale patterning. *Nature Protocols*.

[17] Nguyen T. V., Ukairo O., Khetani S. R. (2015). Establishment of a hepatocyte-kupffer cell coculture model for assessment of proinflammatory cytokine effects on metabolizing enzymes and drug transporters. *Drug Metabolism and Disposition*.

[18] Khetani S. R., Kanchagar C., Ukairo O. (2013). Use of micropatterned cocultures to detect compounds that cause drug-induced liver injury in humans. *Toxicological Sciences*.

[19] Chen M., Zhang J., Wang Y. (2013). The liver toxicity knowledge base: a systems approach to a complex end point. *Clinical Pharmacology and Therapeutics*.

[20] Krzyzewski S., Khetani S. R., Barros S. (2011). Assessing chronic toxicity of fialuridine in a micropatterned hepatocyte co-culture model. *The Toxicologist, Supplement to Toxicological Sciences*.

[21] McKenzie R., Fried M. W., Sallie R. (1995). Hepatic failure and lactic acidosis due to fialuridine (FIAU), an investigational nucleoside analogue for chronic hepatitis B. *The New England Journal of Medicine*.

[22] Manning F. J., Swartz M. (1995). *Review of the Fialuridine (FIAU) Clinical Trials*.

[23] Zinchenko Y. S., Schrum L. W., Clemens M., Coger R. N. (2006). Hepatocyte and Kupffer cells co-cultured on micropatterned surfaces to optimize hepatocyte function. *Tissue Engineering*.

[24] Cho C. H., Park J., Tilles A. W., Berthiaume F., Toner M., Yarmush M. L. (2010). Layered patterning of hepatocytes in co-culture systems using microfabricated stencils. *BioTechniques*.

[25] Liu Y., Wei J., Lu J., Lei D., Yan S., Li X. (2016). Micropatterned coculture of hepatocytes on electrospun fibers as a potential *in vitro* model for predictive drug metabolism. *Materials Science and Engineering: C*.

[26] Gerbal-Chaloin S., Funakoshi N., Caillaud A., Gondeau C., Champon B., Si-Tayeb K. (2014). Human induced pluripotent stem cells in hepatology: beyond the proof of concept. *American Journal of Pathology*.

[27] Ware B. R., Berger D. R., Khetani S. R. (2015). Prediction of drug-induced liver injury in micropatterned co-cultures containing iPSC-derived human hepatocytes. *Toxicological Sciences*.

[28] Ramaiahgari S. C., den Braver M. W., Herpers B. (2014). A 3D in vitro model of differentiated HepG2 cell spheroids with improved liver-like properties for repeated dose high-throughput toxicity studies. *Archives of Toxicology*.

[29] Gunness P., Mueller D., Shevchenko V., Heinzle E., Ingelman-Sundberg M., Noor F. (2013). 3D organotypic cultures of human heparg cells: a tool for in vitro toxicity studies. *Toxicological Sciences*.

[30] Mueller D., Krämer L., Hoffmann E., Klein S., Noor F. (2014). 3D organotypic HepaRG cultures as in vitro model for acute and repeated dose toxicity studies. *Toxicology in Vitro*.

[31] Kostadinova R., Boess F., Applegate D. (2013). A long-term three dimensional liver co-culture system for improved prediction of clinically relevant drug-induced hepatotoxicity. *Toxicology and Applied Pharmacology*.

[32] Gieseck R. L., Hannan N. R. F., Bort R. (2014). Maturation of induced pluripotent stem cell derived hepatocytes by 3D-culture. *PLoS ONE*.

[33] Rebelo S. P., Costa R., Silva M. M., Marcelino P., Brito C., Alves P. M. (2015). Three-dimensional co-culture of human hepatocytes and mesenchymal stem cells: improved functionality in long-term bioreactor cultures. *Journal of Tissue Engineering and Regenerative Medicine*.

[34] Bell C. C., Hendriks D. F. G., Moro S. M. L. (2016). Characterization of primary human hepatocyte spheroids as a model system for drug-induced liver injury, liver function and disease. *Scientific Reports*.

[35] Tong W. H., Fang Y., Yan J. (2016). Constrained spheroids for prolonged hepatocyte culture. *Biomaterials*.

[36] Messner S., Agarkova I., Moritz W., Kelm J. M. (2013). Multi-cell type human liver microtissues for hepatotoxicity testing. *Archives of Toxicology*.

[37] Miyamoto Y., Ikeuchi M., Noguchi H., Yagi T., Hayashi S. (2015). Spheroid formation and evaluation of hepatic cells in a three-dimensional culture device. *Cell Medicine*.

[38] Takayama K., Kawabata K., Nagamoto Y. (2013). 3D spheroid culture of hESC/hiPSC-derived hepatocyte-like cells for drug toxicity testing. *Biomaterials*.

[39] Ma X., Qu X., Zhu W. (2016). Deterministically patterned biomimetic human iPSC-derived hepatic model via rapid 3D bioprinting. *Proceedings of the National Academy of Sciences of the United States of America*.

[40] Li C. Y., Stevens K. R., Schwartz R. E., Alejandro B. S., Huang J. H., Bhatia S. N. (2014). Micropatterned cell-cell interactions enable functional encapsulation of primary hepatocytes in hydrogel microtissues. *Tissue Engineering Part A*.

[41] Lutolf M. P., Hubbell J. A. (2005). Synthetic biomaterials as instructive extracellular microenvironments for morphogenesis in tissue engineering. *Nature Biotechnology*.

[42] Tasnim F., Toh Y., Qu Y. (2016). Functionally enhanced human stem cell derived hepatocytes in galactosylated cellulosic sponges for hepatotoxicity testing. *Molecular Pharmaceutics*.

[43] Jungermann K., Kietzmann T. (1996). Zonation of parenchymal and nonparenchymal metabolism in liver. *Annual Review of Nutrition*.

[44] Allen J. W., Bhatia S. N. (2003). Formation of steady-state oxygen gradients in vitro: application to liver zonation. *Biotechnology and Bioengineering*.

[45] Allen J. W., Khetani S. R., Bhatia S. N. (2005). In vitro zonation and toxicity in a hepatocyte bioreactor. *Toxicological Sciences*.

[46] Novik E., Maguire T. J., Chao P., Cheng K. C., Yarmush M. L. (2010). A microfluidic hepatic coculture platform for cell-based drug metabolism studies. *Biochemical Pharmacology*.

[47] Sivaraman A., Leach J. K., Townsend S. (2005). A microscale in vitro physiological model of the liver: predictive screens for drug metabolism and enzyme induction. *Current Drug Metabolism*.

[48] Esch M. B., Prot J.-M., Wang Y. I. (2015). Multi-cellular 3D human primary liver cell culture elevates metabolic activity under fluidic flow. *Lab on a Chip*.

[49] Lee P. J., Hung P. J., Lee L. P. (2007). An artificial liver sinusoid with a microfluidic endothelial-like barrier for primary hepatocyte culture. *Biotechnology and Bioengineering*.

[50] Miller P. G., Shuler M. L. (2016). Design and demonstration of a pumpless 14 compartment microphysiological system. *Biotechnology and Bioengineering*.

[51] Kobayashi A., Yamakoshi K., Yajima Y., Utoh R., Yamada M., Seki M. (2013). Preparation of stripe-patterned heterogeneous hydrogel sheets using microfluidic devices for high-density coculture of hepatocytes and fibroblasts. *Journal of Bioscience and Bioengineering*.

[52] Skardal A., Devarasetty M., Soker S., Hall A. R. (2015). In situ patterned micro 3D liver constructs for parallel toxicology testing in a fluidic device. *Biofabrication*.

[53] Ma C., Zhao L., Zhou E.-M., Xu J., Shen S., Wang J. (2016). On-chip construction of liver lobule-like microtissue and its application for adverse drug reaction assay. *Analytical Chemistry*.

[54] Bhise N. S., Manoharan V., Massa S. (2016). A liver-on-a-chip platform with bioprinted hepatic spheroids. *Biofabrication*.

[55] Bavli D., Prill S., Ezra E. (2016). Real-time monitoring of metabolic function in liver-on-chip microdevices tracks the dynamics of mitochondrial dysfunction. *Proceedings of the National Academy of Sciences of the United States of America*.

[56] Rennert K., Steinborn S., Gröger M. (2015). A microfluidically perfused three dimensional human liver model. *Biomaterials*.

[57] Vernetti L. A., Senutovitch N., Boltz R. (2016). A human liver microphysiology platform for investigating physiology, drug safety, and disease models. *Experimental Biology and Medicine*.

[58] Viravaidya K., Sin A., Shuler M. L. (2004). Development of a microscale cell culture analog to probe naphthalene toxicity. *Biotechnology Progress*.

[59] Choucha-Snouber L., Aninat C., Grsicom L. (2013). Investigation of ifosfamide nephrotoxicity induced in a liver-kidney co-culture biochip. *Biotechnology and Bioengineering*.

[60] Materne E.-M., Ramme A. P., Terrasso A. P. (2015). A multi-organ chip co-culture of neurospheres and liver equivalents for long-term substance testing. *Journal of Biotechnology*.

[61] Esch M. B., Mahler G. J., Stokol T., Shuler M. L. (2014). Body-on-a-chip simulation with gastrointestinal tract and liver tissues suggests that ingested nanoparticles have the potential to cause liver injury. *Lab on a Chip*.

[62] Wagner I., Materne E.-M., Brincker S. (2013). A dynamic multi-organ-chip for long-term cultivation and substance testing proven by 3D human liver and skin tissue co-culture. *Lab on a Chip*.

[63] Materne E.-M., Maschmeyer I., Lorenz A. K. (2015). The multi-organ chip—a microfluidic platform for long-term multi-tissue coculture. *Journal of Visualized Experiments*.

[64] Sung J. H., Kam C., Shuler M. L. (2010). A microfluidic device for a pharmacokinetic-pharmacodynamic (PK-PD) model on a chip. *Lab on a Chip—Miniaturisation for Chemistry and Biology*.

[65] Maschmeyer I., Lorenz A. K., Schimek K. (2015). A four-organ-chip for interconnected long-term co-culture of human intestine, liver, skin and kidney equivalents. *Lab on a Chip—Miniaturisation for Chemistry and Biology*.

[66] Oleaga C., Bernabini C., Smith A. S. T. (2016). Multi-Organ toxicity demonstration in a functional human *in vitro* system composed of four organs. *Scientific Reports*.

[67] Olinga P., Schuppan D. (2013). Precision-cut liver slices: a tool to model the liver ex vivo. *Journal of Hepatology*.

[68] Van de Bovenkamp M., Groothuis G. M. M., Meijer D. K. F., Olinga P. (2007). Liver fibrosis in vitro: cell culture models and precision-cut liver slices. *Toxicology in Vitro*.

[69] van Midwoud P. M., Merema M. T., Verweij N., Groothuis G. M. M., Verpoorte E. (2011). Hydrogel embedding of precision-cut liver slices in a microfluidic device improves drug metabolic activity. *Biotechnology and Bioengineering*.

[70] van Midwoud P. M., Merema M. T., Verpoorte E., Groothuis G. M. M. (2011). Microfluidics enables small-scale tissue-based drug metabolism studies with scarce human tissue. *Journal of Laboratory Automation*.

[71] Yuet M. K., Zhang J., Zhou S. (2007). Novel intra-tissue perfusion system for culturing thick liver tissue. *Tissue Engineering*.

[72] Schumacher K., Khong Y.-M., Chang S., Ni J., Sun W., Yu H. (2007). Perfusion culture improves the maintenance of cultured liver tissue slices. *Tissue Engineering*.

[73] Yoshizato K., Tateno C. (2013). A mouse with humanized liver as an animal model for predicting drug effects and for studying hepatic viral infection: where to next?. *Expert Opinion on Drug Metabolism and Toxicology*.

[74] Strom S. C., Davila J., Grompe M. (2010). Chimeric mice with humanized liver: tools for the study of drug metabolism, excretion, and toxicity. *Methods in Molecular Biology*.

[75] Sanoh S., Horiguchi A., Sugihara K. (2012). Prediction of in vivo hepatic clearance and half-life of drug candidates in human using chimeric mice with humanized liver. *Drug Metabolism and Disposition*.

[76] Sanoh S., Ohta S. (2014). Chimeric mice transplanted with human hepatocytes as a model for prediction of human drug metabolism and pharmacokinetics. *Biopharmaceutics and Drug Disposition*.

[77] Yamada T., Okuda Y., Kushida M. (2014). Human hepatocytes support the hypertrophic but not the hyperplastic response to the murine nongenotoxic hepatocarcinogen sodium phenobarbital in an *in vivo* study using a chimeric mouse with humanized liver. *Toxicological Sciences*.

[78] Xu D., Nishimura T., Nishimura S. (2014). Fialuridine induces acute liver failure in chimeric TK-NOG mice: a model for detecting hepatic drug toxicity prior to human testing. *PLoS Medicine*.

[79] Xu D., Wu M., Nishimura S. (2015). Chimeric TK-NOG mice: a predictive model for cholestatic human liver toxicity. *Journal of Pharmacology and Experimental Therapeutics*.

[80] Kakuni M., Morita M., Matsuo K. (2012). Chimeric mice with a humanized liver as an animal model of troglitazone-induced liver injury. *Toxicology Letters*.

[81] Akkina R. (2013). Human immune responses and potential for vaccine assessment in humanized mice. *Current Opinion in Immunology*.

[82] Wilson E. M., Bial J., Tarlow B. (2014). Extensive double humanization of both liver and hematopoiesis in FRGN mice. *Stem Cell Research*.

[83] Bility M. T., Zhang L., Washburn M. L., Curtis T. A., Kovalev G. I., Su L. (2012). Generation of a humanized mouse model with both human immune system and liver cells to model hepatitis C virus infection and liver immunopathogenesis. *Nature Protocols*.

[84] Chen A. A., Thomas D. K., Ong L. L., Schwartz R. E., Golub T. R., Bhatia S. N. (2011). Humanized mice with ectopic artificial liver tissues. *Proceedings of the National Academy of Sciences of the United States of America*.

[85] Ohashi K., Marion P. L., Nakai H. (2000). Sustained survival of human hepatocytes in mice: a model for in vivo infection with human hepatitis B and hepatitis delta viruses. *Nature Medicine*.

[86] Persson M., Løye A. F., Jacquet M. (2014). High-content analysis/screening for predictive toxicology: application to hepatotoxicity and genotoxicity. *Basic and Clinical Pharmacology and Toxicology*.

[87] Wink S., Hiemstra S., Huppelschoten S. (2014). Quantitative high content imaging of cellular adaptive stress response pathways in toxicity for chemical safety assessment. *Chemical Research in Toxicology*.

[88] Tolosa L., Gómez-Lechón M. J., Donato M. T. (2015). High-content screening technology for studying drug-induced hepatotoxicity in cell models. *Archives of Toxicology*.

[89] O'Brien P. J., Irwin W., Diaz D. (2006). High concordance of drug-induced human hepatotoxicity with in vitro cytotoxicity measured in a novel cell-based model using high content screening. *Archives of Toxicology*.

[90] Garside H., Marcoe K. F., Chesnut-Speelman J. (2014). Evaluation of the use of imaging parameters for the detection of compound-induced hepatotoxicity in 384-well cultures of HepG2 cells and cryopreserved primary human hepatocytes. *Toxicology in Vitro*.

[91] Trask O. J., Moore A., LeCluyse E. L. (2014). A micropatterned hepatocyte coculture model for assessment of liver toxicity using high-content imaging analysis. *Assay and Drug Development Technologies*.

[92] Cole S. D., Madren-Whalley J. S., Li A. P., Dorsey R., Salem H. (2014). High content analysis of an in vitro model for metabolic toxicity: results with the model toxicants 4-aminophenol and cyclophosphamide. *Journal of Biomolecular Screening*.

[93] Sirenko O., Hesley J., Rusyn I., Cromwell E. F. (2014). High-content assays for hepatotoxicity using induced pluripotent stem cell-derived cells. *Assay and Drug Development Technologies*.

[94] Grimm F. A., Iwata Y., Sirenko O., Bittner M., Rusyn I. (2015). High-content assay multiplexing for toxicity screening in induced pluripotent stem cell-derived cardiomyocytes and hepatocytes. *Assay and Drug Development Technologies*.

[95] Pradip A., Steel D., Jacobsson S. (2016). High content analysis of human pluripotent stem cell derived hepatocytes reveals drug induced steatosis and phospholipidosis. *Stem Cells International*.

[96] Godoy P., Bolt H. M. (2012). Toxicogenomic-based approaches predicting liver toxicity in vitro. *Archives of Toxicology*.

[97] Zhang M., Chen M., Tong W. (2012). Is toxicogenomics a more reliable and sensitive biomarker than conventional indicators from rats to predict drug-induced liver injury in humans?. *Chemical Research in Toxicology*.

[98] Rodrigues R. M., Heymans A., De Boe V. (2016). Toxicogenomics-based prediction of acetaminophen-induced liver injury using human hepatic cell systems. *Toxicology Letters*.

[99] Schug M., Heise T., Bauer A. (2008). Primary rat hepatocytes as in vitro system for gene expression studies: comparison of sandwich, Matrigel and 2D cultures. *Archives of Toxicology*.

[100] Jiang J., Wolters J. E. J., van Breda S. G., Kleinjans J. C., de Kok T. M. (2015). Development of novel tools for the in vitro investigation of drug-induced liver injury. *Expert Opinion on Drug Metabolism and Toxicology*.

[101] Iruzubieta P., Arias-Loste M. T., Barbier-Torres L., Martinez-Chantar M. L., Crespo J. (2015). The need for biomarkers in diagnosis and prognosis of drug-induced liver disease: does metabolomics have any role?. *BioMed Research International*.

[102] Raies A. B., Bajic V. B. (2016). In silico toxicology: computational methods for the prediction of chemical toxicity. *Wiley Interdisciplinary Reviews: Computational Molecular Science*.

[103] Zhu X.-W., Sedykh A., Liu S.-S. (2014). Hybrid *in silico* models for drug-induced liver injury using chemical descriptors and *in vitro* cell-imaging information. *Journal of Applied Toxicology*.

[104] Mulliner D., Schmidt F., Stolte M., Spirkl H.-P., Czich A., Amberg A. (2016). Computational models for human and animal hepatotoxicity with a global application scope. *Chemical Research in Toxicology*.

[105] Vedani A., Dobler M., Hu Z., Smieško M. (2015). OpenVirtualToxLab—a platform for generating and exchanging in silico toxicity data. *Toxicology Letters*.

[106] Kleinstreuer N. C., Yang J., Berg E. L. (2014). Phenotypic screening of the ToxCast chemical library to classify toxic and therapeutic mechanisms. *Nature Biotechnology*.

[107] Shoda L. K. M., Woodhead J. L., Siler S. Q., Watkins P. B., Howell B. A. (2014). Linking physiology to toxicity using DILIsym®, a mechanistic mathematical model of drug-induced liver injury. *Biopharmaceutics and Drug Disposition*.

[108] Howell B. A., Yang Y., Kumar R. (2012). In vitro to in vivo extrapolation and species response comparisons for drug-induced liver injury (DILI) using DILIsymTM: a mechanistic, mathematical model of DILI. *Journal of Pharmacokinetics and Pharmacodynamics*.

[109] Subramanian K., Raghavan S., Bhat A. R. (2008). A systems biology based integrative framework to enhance the predictivity of *in vitro* methods for drug-induced liver injury. *Expert Opinion on Drug Safety*.

[110] Shardlow C. E., Generaux G. T., Patel A. H., Tai G., Tran T., Bloomer J. C. (2013). Impact of physiologically based pharmacokinetic modeling and simulation in drug development. *Drug Metabolism and Disposition*.

[111] Zurlinden T. J., Reisfeld B. (2016). Physiologically based modeling of the pharmacokinetics of acetaminophen and its major metabolites in humans using a Bayesian population approach. *European Journal of Drug Metabolism and Pharmacokinetics*.

[112] LeCluyse E. L., Witek R. P., Andersen M. E., Powers M. J. (2012). Organotypic liver culture models: meeting current challenges in toxicity testing. *Critical Reviews in Toxicology*.

[113] Esch M. B., King T. L., Shuler M. L. (2011). The role of body-on-a-chip devices in drug and toxicity studies. *Annual Review of Biomedical Engineering*.

[114] Rawlins M. D. (2004). Cutting the cost of drug development?. *Nature Reviews Drug Discovery*.

[115] Kaitin K. I. (2010). Deconstructing the drug development process: the new face of innovation. *Clinical Pharmacology and Therapeutics*.

[116] Berger D. R., Ware B. R., Davidson M. D., Allsup S. R., Khetani S. R. (2015). Enhancing the functional maturity of induced pluripotent stem cell-derived human hepatocytes by controlled presentation of cell-cell interactions *in vitro*. *Hepatology*.

[117] Lin C., Shi J., Moore A., Khetani S. R. (2016). Prediction of drug clearance and drug-drug interactions in microscale cultures of human hepatocytes. *Drug Metabolism and Disposition*.

[118] Xu D., Peltz G. (2016). Can humanized mice predict drug ‘behavior’ in humans?. *Annual Review of Pharmacology and Toxicology*.

